# Regional variations in the management of primary hyperparathyroidism in Sweden: population-based case-control study

**DOI:** 10.1093/bjsopen/zrad154

**Published:** 2024-02-07

**Authors:** David Thorsteinsson, Fredrik Granath, Robert Bränström, Anna Koman, Jan Zedenius, Inga-Lena Nilsson

**Affiliations:** Department of Breast, Endocrine Tumours and Sarcoma, Karolinska University Hospital, Stockholm, Sweden; Department of Molecular Medicine and Surgery, Karolinska Institutet, Stockholm, Sweden; Department of Medicine Solna, Division of Clinical Epidemiology, Karolinska Institutet, Stockholm, Sweden; Department of Breast, Endocrine Tumours and Sarcoma, Karolinska University Hospital, Stockholm, Sweden; Department of Molecular Medicine and Surgery, Karolinska Institutet, Stockholm, Sweden; Department of Breast, Endocrine Tumours and Sarcoma, Karolinska University Hospital, Stockholm, Sweden; Department of Molecular Medicine and Surgery, Karolinska Institutet, Stockholm, Sweden; Department of Breast, Endocrine Tumours and Sarcoma, Karolinska University Hospital, Stockholm, Sweden; Department of Molecular Medicine and Surgery, Karolinska Institutet, Stockholm, Sweden; Department of Breast, Endocrine Tumours and Sarcoma, Karolinska University Hospital, Stockholm, Sweden; Department of Molecular Medicine and Surgery, Karolinska Institutet, Stockholm, Sweden

## Abstract

**Background:**

Substantial disparities in the utilization of parathyroidectomy for primary hyperparathyroidism have been reported. This study aimed to analyse regional variations in parathyroidectomy incidence with respect to the patient's disease burden and socioeconomic status.

**Methods:**

A population-based case-control study included all patients with primary hyperparathyroidism who underwent parathyroidectomy in Sweden between 2008 and 2017 and 10 matched controls. Data on demographic and socioeconomic variables, co-morbidities and drug prescriptions were collected from relevant national registers. Conditional logistic regression was used to analyse predictors of parathyroidectomy.

**Results:**

A total of 8626 patients with primary hyperparathyroidism (77% women) underwent parathyroidectomy during the study interval. The annual incidence of parathyroidectomy was 9.0 per 100 000 persons. The annual age-adjusted regional incidences of parathyroidectomy varied between 3.3 and 16.9 operations per 100 000 inhabitants. Except for a small underrepresentation of patients with lower education, no effect of socioeconomic variables was observed. Compared with matched controls, the parathyroidectomy group had increased odds ratios of having developed classical symptoms of primary hyperparathyroidism and being prescribed medication against cardiovascular disorders and psychiatric illness at the time of parathyroidectomy. Increased risks of kidney stones and osteoporosis were observed 5 years before parathyroidectomy. Patients with primary hyperparathyroidism selected for parathyroidectomy from regions with a low incidence of operations had a higher prevalence of kidney stones, osteoporosis and hypertension, as well as larger adenomas and higher calcium levels at the time of parathyroidectomy compared with patients in high-incidence regions.

**Conclusion:**

The considerable variation in parathyroidectomy seems more likely associated with different clinical thresholds for detection of primary hyperparathyroidism and referral to surgery than socioeconomic disparities.

## Teaser

A Swedish population-based case-control study on regional differences in referring patients for parathyroidectomy and socioeconomic and medical predictors of surgery.

## Introduction

Primary hyperparathyroidism (PHPT) is a common endocrine disorder with a rising prevalence^1,2^ and a single curative treatment: parathyroidectomy (PTX). The disease classically manifests through skeletal and renal disease, and there is reasonable consensus on managing PHPT with these manifestations^3–6^. The impact of treatment in biochemically milder forms of PHPT remains controversial, especially regarding the causal connection to co-morbidities and risks of fractures and cardiovascular complications^7–10^. Despite clear clinical guidelines regarding symptomatic PHPT and asymptomatic PHPT in young patients and patients with high calcium levels, the benefits of PTX compared with simple observation are not well established^11^. Large differences in the management of PHPT in countries with universal healthcare similar to Sweden are reported^12–14^ as well as underutilization of PTX in the elderly population^15^. Furthermore, regional differences in healthcare utilization in Sweden are of a considerable magnitude and largely unexplained by supply and demand^16,17^. The management of the Swedish healthcare system is decentralized to the country’s 21 semi-autonomous regions, which are regulated by the Health and Medical Services Act (2017:30). Most patients with PHPT are diagnosed in primary care. Based on earlier screening studies, the natural incidence of PHPT can be assumed to be similar across the country, with all regions offering specialized care for PHPT. The study objective was to analyse potential regional variations in the incidence of PTX with respect to disease burden as measured by the prevalence and distribution of co-morbidities. Furthermore, the aim was to examine if socioeconomic factors and healthcare supply would explain this suspected variation.

## Methods

Personal identity numbers were used for linkage between different national population registers: The National Patient Register (NPR), The Longitudinal Integrated Database for Health Insurance and Labour Market Research (LISA) and National Prescribed Drug Register (NPDR). In this population-based case-control study, all patients operated on with PTX for PHPT in Sweden, between 1 January 2008 and 31 December 2017, were identified and included by use of Swedish personal identity numbers through The Scandinavian Register for Thyroid, Parathyroid, and Adrenal Surgery (SQRTPA) and the Swedish National Cancer Register (SCR). SQRTPA was started in 2004 and is a well validated nationwide Swedish quality register for endocrine surgical procedures^17^. SQRTPA includes information on patients with PTX, including the date of diagnosis and PTX, preoperative calcium levels (mmol/l) and adenoma weights (g). SCR covers the entire population and has, since its introduction in 1958, also included some benign tumours such as parathyroid adenomas. It is compulsory, by law, for healthcare providers in Sweden to report to the SCR, and underreporting has been estimated to be at most 4%^18^. NPR includes all tertiary diagnostic and procedural codes (ICD-10 and International Classification of Health Interventions (ICHI), respectively) for Sweden since 1984, and external validation has shown good reliability^18^. NPR also includes data on healthcare consumption measured as the number of visits to both in- and outpatient tertiary clinics and dates of admittance and discharge. LISA provides information on individual and total family income (as reported by the Income and Taxation Register in Swedish krona (SEK)) the year before the date of index and level of education (described on a scale of 1–7; redefined as primary [1–2], secondary [2–3] and tertiary education [4–7]). SEK was converted to Euro using the mean currency exchange rate for the study period (9.45 SEK = 1 Euro) as reported by the Swedish Statistics Agency (SCB). NPDR includes data on all filled prescription medication, defined by the Anatomical Therapeutic Chemical (ATC) classification, dispensed by pharmacies since July 2005^19^. *Table S1* shows the reported ICD-10 and ATC index codes.

The SCB selected and matched the controls through risk-set sampling. The index date was defined as the date of PTX, and controls were matched according to age, sex and regional address on 31 December of the year before the index date. Ten controls were selected for each case. In addition to matching variables, SCB data included country of birth and date of death for patients and controls. Variables from SQRTPA, SCB, NPR and LISA were pooled in an anonymized database with unique numbering for individuals as well as for matched sets with the removal of duplicates. The study size thus included all operated on individuals in the study interval with 10 matched controls for each patient. Population counts by sex, 5-year age categories and the region of residence were acquired for every study year through SCB to allow a population-based calculation of crude regional incidence of PTX calculated as a sum of cases operated on in the county over the whole interval divided by the sum of the source population. Data collection and linkage were done in December 2020.

Descriptive summary statistics were used to show counts (medians and interquartile range (i.q.r.) or means and standard deviations (s.d.)) and proportions for discreet and continuous variables. Incidence risk ratios (IRR) for each region were calculated and adjusted for distribution of sex, age and year of PTX by Poisson regression with the Region of Stockholm, the largest region, as the baseline. Conditional logistic regression was used for matched sets when comparing patients with controls. Case status was dichotomized and used as the dependent variable, with age, sex, county of residence and year of PTX as independent predictors. Age and sex are confounders controlled for through matching and regression. Results are shown as odds ratio (OR) with 95% c.i. (the α-level was set at 0.05). After grouping, a conditional logistic regression model was used to analyse ATC and ICD codes. Because the NPR does not include data from primary care, we have introduced a risk of information bias in our study. To address this bias for diagnoses that do not require specialized care and would thus run a chance of being overrepresented in the case group, we used an either/or function to register a diagnosis for each row through either the ICD or ATC code. Thus, a person in the study population was considered to have a diagnosis by either being registered with an ICD code in the NPR or by the relevant ATC code in NPDR. Summary statistics were then shown for each group for 5 years and 1 year before surgery. Pearson’s chi-square test was used to analyse frequency differences in contingency tables; the *t*-test or Mann–Whitney *U* test was used to compare patient characteristics. Results are presented as mean and standard deviations (s.d.), or median and range dependent on data distribution. The study report follows guidelines provided by the STROBE statement for observational studies^20^. Missing data were reported, and a complete case analysis was performed for each variable if necessary. Multiple imputation with chained equations (MICE) was done using the MICE package for R^21^ with predictive mean matching for preoperative calcium levels and adenoma weights, where sex, age, co-morbidities and co-medication were included in the prediction model. Statistical calculations, graphs and maps were done using R studio version 2021.09.2 + 382 (Boston, MA, USA) with Tidyverse package version 1.3.1^22^, and multiple logistic regression was performed with the Survival package 3.4–0^23^. Ethical approval was obtained before data collection (Dnr. 2019-02149).

## Results

### Study participants and socioeconomic characteristics

**Fig. 1:**
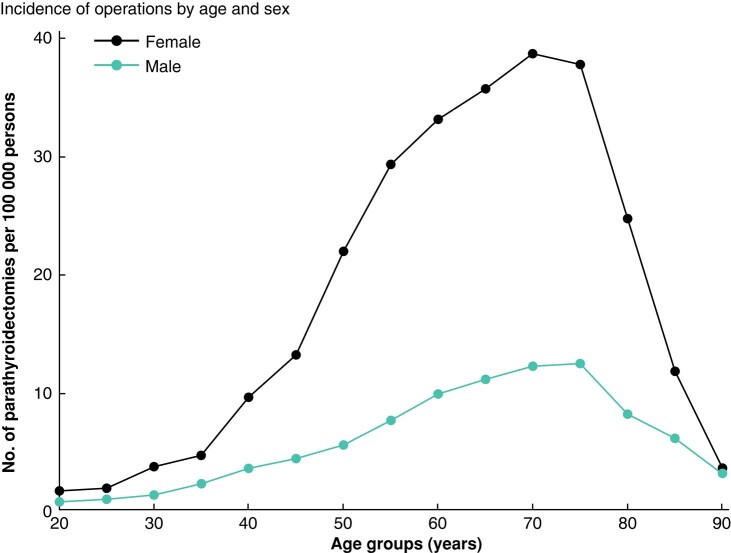
Incidence of parathyroidectomies by age and sex

**Fig. 2:**
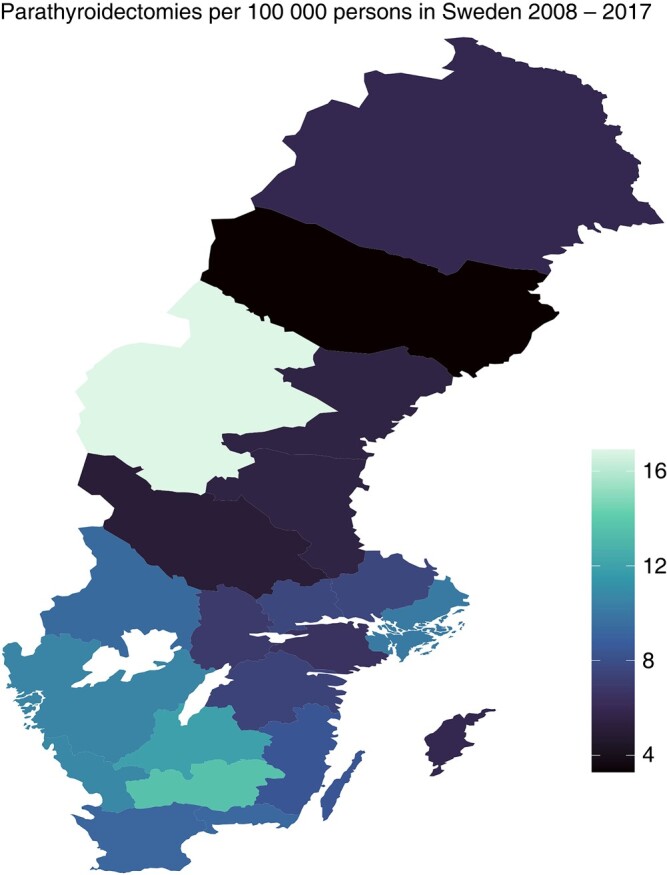
Incidence of parathyroidectomies by region in Sweden Range from 3.3 (dark) to 16.9 (light) parathyroidectomies per 100 000 persons.

A total of 8626 patients with PHPT subjected to PTX in Sweden during the study interval were identified. The median age was 63 years (range 12–96 years; i.q.r. 18 years), and 77% (*n* = 6633) were female. The distributions of sex, age, education, income and country of birth for patients and controls are shown in *Table 1*. The overall PTX incidence during the study interval was 9.0 operations per 100 000 persons per year. Age- and sex-specific incidences are shown in *Fig. 1*. We observed a considerable difference in age-adjusted PTX incidence between regions, ranging from 3.3 to 16.9 PTX per 100 000 persons, as shown in *Fig. 2*. The education, income and ethnicity of PTX patients were compared with the matched control sample. Persons with only primary education level were slightly underrepresented among patients compared with persons having secondary and tertiary education, OR 0.93 (95% c.i. 0.88 to 0.99) and OR 0.91 (95% c.i. 0.86 to 0.98) respectively. No difference in income was observed between patients and controls. Persons born in Africa were overrepresented among patients compared with persons of Nordic origin, OR 1.44 (95% c.i. 1.15 to 1.81), while no other significant differences concerning the country of birth were observed.

**Table 1:** Characteristics of patients and controls

Characteristics	Controls (*n* = 86 260)	Patients (*n* = 8626)
Women	66 330 (77)	6633 (77)
Age (years), median (i.q.r.)	63 (18)	63 (18)
**Education**		
Primary	21 688 (25.1)	2073 (24.0)
Secondary	36 875 (42.8)	3732 (43.3)
Tertiary	26 259 (30.4)	2711 (31.4)
Missing	1438 (1.7)	110 (1.3)
**Ethnicity**		
Nordic countries	76 000 (88.1)	7640 (88.6)
Africa	741 (0.9)	102 (1.2)
Asia	3283 (3.8)	295 (3.4)
Europe	5428 (6.3)	498 (5.8)
Americas	791 (0.9)	90 (1.0)
Oceania	15 (0.0)	1 (0.0)
Unknown	2 (0.0)	0 (0.0)
**Taxed income (€)**		
Individual, mean(s.d.)	238(373)	241(346)
Missing	237	13
Family, mean(s.d.)	429(238)	432(241)
Missing	237	13

Values are *n* (%) unless otherwise stated.

### Co-morbidities and prescribed medications

*Table 2* shows the number and proportions of patients selected for PTX and fulfilling established eligibility criteria for PTX by region (79 and 68% of patients in regions with low and high incidence of PTX respectively (*P* < 0.001)). *Table S2* lists the prevalence of co-morbidities and medication among PTX patients 1 year and 5 years before surgery, and these are compared with the corresponding prevalences among matched population controls.

**Table 2:** Patients selected for parathyroidectomy meeting established surgery criteria

Variable	Low-incidence regions	High-incidence regions	*P* (chi^2^)	Total
Calcium ≥1.45 mmol/l	613 (57.8)	2802 (44.4)	**< 0.001**	3415 (46.3)‡
Age < 50 years	200 (17.2)	1325 (17.7)	0.349	1525 (17.7)
Kidney stones*	166 (14.3)	633 (8.5)	**< 0.001**	799 (9.3)
Osteoporosis†	167 (14.4)	1065 (14.3)	0.472	1232 (14.3)
Any criteria above	866 (78.7)	4551 (68.1)	**< 0.001**	5417 (73.5)‡

Values are *n* (%) unless otherwise stated. *Registered diagnosis within 5 years before parathyroidectomy. †Diagnosis and/or expedited prescriptions of bisphosphonates. ‡Denominator = 7372 (not missing). Bold indicates *P* < 0.05.

The prevalence of co-morbidities among patients with PHPT selected for PTX was high. Within the year before PTX, 45.4% were registered with hypertension, 12% had osteoporosis and 5.9% had kidney stones. Antacids were prescribed to 25% and anxiolytics to 20%. Compared with controls, the prevalence of kidney stones, kidney failure and osteoporosis was higher 5 years before PTX. Stroke was overrepresented in the PTX group, but no difference was found for myocardial infarction or antidiabetic medications. The OR for pancreatitis was significantly increased 5 years and 1 year before PTX.

### Comparison between low- and high-incidence regions

**Fig. 3:**
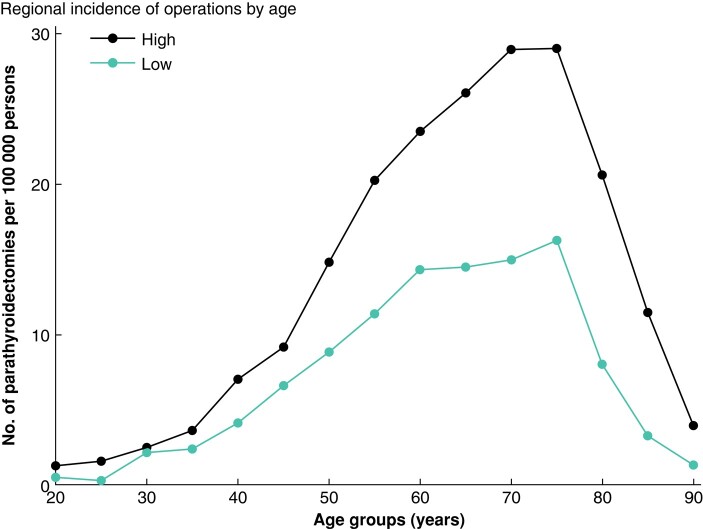
Incidence of parathyroidectomies by region

The age distribution by low- and high-incidence regions is shown in *Fig. 3*. The relative differences between low- and high-incidence regions remained relatively constant during the study interval. No interaction between incidence group and year of surgery was observed (*P* = 0.210). Differences were more pronounced among patients above 50 years of age.

*Table 3* shows the higher likelihood of kidney failure, kidney stones, osteoporosis, hypertension and non-steroidal anti-inflammatory drug (NSAID) use among patients with PHPT selected for PTX in regions with a low incidence of PTX. The incidence of PTX grouped the regions into low-incidence (adjusted incidence <7 PTX per 100 000 population, eight regions, *n* = 1161) and high-incidence regions (>7 per 100 000 population, 13 regions, *n* = 7465). The differences were noticeable 5 years before PTX but were highest within the year before the index date.

**Table 3:** Comparison between low- and high-incidence regions

ICD and/or ATC codes	Low-incidence region (*n* = 1161)			High-incidence region (*n* = 7465)		
	OR (95% c.i.)	Patients (%)	Controls (%)	OR (95% c.i.)	Patients (%)	Controls (%)
Kidney stones	36.3 (24.3,54.4)	9.5	0.28	17.4 (14.8,20.5)	5.3	0.3
Musculoskeletal diagnoses	2.1 (1.8,2.5)	17.3	9.1	1.7 (1.6,1.9)	16.1	10
Osteoporosis medication	4.9 (3.8,6.3)	9.0	2.2	2.7 (2.4,2.9)	7.1	3.0
Osteoporosis diagnosis and/or medication	6.7 (5.3,8.4)	12.7	2.4	4.4 (4.0,4.8)	11.9	3.3
NSAID medications	2.2 (1.9,2.5)	29.7	16.6	1.6 (1.5,1.7)	25.6	17.8
Hypertension diagnosis	10.7 (9.1,12.6)	35.3	5.9	8.0 (7.5,8.6)	29.2	5.7
**Adenoma weight, percentiles**						
25th	137 (17)			1118 (24)		
50th	209 (27)			1195 (26)		
75th	198 (25)			1170 (25)		
100th	244 (31)			1132 (25)		
Missing	373			2850		
Median (range), g	0.49 (0.06–8.22)			0.43 (0.02–8.40)		
**Ionized calcium concentration, percentiles**						
25th	196 (18)			1816 (29)		
50th	307 (29)			1994 (31)		
75th	263 (25)			1336 (21)		
100th	300 (28)			1215 (19)		
Missing	95			1104		
Median (range), mmol/l	1.46 (1.17–2.58)			1.44 (1.14–2.62)		

Values are *n* (%) unless otherwise stated. ICD, international classification of disease; ATC, anatomical therapeutic chemical classification; NSAID, non-steroidal anti-inflammatory drugs.

Patients in the low-incidence regions had higher preoperative calcium concentrations (mean 1.48(0.12) *versus* 1.45(0.12) mmol/l, *P* < 0.001). A total of 63% (*n* = 5400) had information on adenoma weight, as shown in *Table 3*; patients in low-incidence regions had larger adenomas represented by a significantly higher percentage of patients with adenoma weight over the 75th percentile compared with high-incidence regions. These differences remained after multiple imputation with estimated mean calcium 1.48(0.003) mmol/l and 1.45(0.004) mmol/l and estimated mean adenoma weight 0.68(0.02) g and 0.59(0.02) g for regions with low and high incidence of PTX respectively (*P* < 0.0001 for calcium and *P* < 0.0005 for adenoma weight).

Patients of African ethnicity had larger adenomas than non-Africans (median 0.54 g *versus* 0.43 g) and were overrepresented in high-incidence regions. There was no significant difference in income between patients and controls in their respective region. Patients with secondary and tertiary education in high-incidence regions had a slightly higher odds of being operated on (OR 1.08 (1.02 to 1.15) and OR 1.10 (1.03 to 1.18) respectively) compared with patients with primary education. *Table S3* shows socioeconomic factors for patients in high- and low-incidence regions.

To estimate the general access to healthcare, the prevalence of co-morbidities and drug prescriptions in control groups was compared. This gives an estimate of differences in care-seeking behaviours for regions with a low and high incidence of operations. As seen in *Table 3*, the controls in the high-incidence regions had a higher prevalence of all diagnoses except hypertension, with the most considerable difference for NSAID use, 1.2%. The median waiting time from the first diagnosis to the date of surgery varied between 208 (i.q.r. 103–462) days in low-incidence regions and 189 (i.q.r. 93–399) days in high-incidence areas (*P* = 0.029). Five years before the index date, the number of visits to outpatient clinics was higher in high-incidence regions (37% *versus* 32% had at least seven visits, *P* < 0.001). Still, the frequency of inpatient care was similar (1 (i.q.r. 0–1)) in both groups.

## Discussion

The main finding of this study was the considerable regional variation in the incidence of PTX in Sweden. The age-adjusted incidence varied between regions by a factor of six (between 3.33 and 16.9 PTX per 100 000 persons). Another important finding was the high prevalence of co-morbidities among patients with PHPT selected for PTX in low-incidence regions compared with high-incidence regions, as well as a higher disease burden in the whole PTX patient group compared with matched controls. As expected, the prevalence of kidney disorders and osteoporosis was high. This risk was as high as 4–5-fold compared with the controls despite mild hypercalcaemia in most cases. The increased prevalence of classical symptoms was already evident 5 years before PTX. In the 5 years before PTX, hypertension (combined prescriptions and diagnoses) doubled in prevalence from 24 to 45%. Distal long bone fractures were twice as likely in the PTX group, with 2% of patients having experienced a fracture in the year before surgery. The regional differences reported here are not supposed to reflect regional difference in prevalence of PHPT. Based on earlier population-based surveys from different parts of Sweden, the prevalence of PHPT is estimated to be about 1% among adults, higher among women and with increasing prevalence with advancing age reaching about 3% for women above 60 years of age, of which only a small fraction was referred to surgery^24–26^. The high incidence observed in region 23, Jämtland, can reflect aggregation of undiagnosed patients in connection with health-oriented research efforts^17^. This study thus confirms a large discrepancy in the regional utilization of PTX in the treatment of PHPT in Sweden. Although socioeconomic factors may contribute to the observed regional difference in the incidence of PTX to a small extent, the primary factors are likely to be a combination of higher regional thresholds for surgical referral and possibly a lower detection of PHPT in regions with low per capita incidence of PTX. This is supported by the fact that patients in the low-incidence regions have a higher prevalence of both classical and non-classical symptoms, higher calcium levels and larger adenoma weights at the time of surgery. Despite relatively mild calcium levels, there was a significant increase in the proportion of these symptoms over 5 years, with a larger increase in regions with a low referral for PTX. Based on the regional differences observed in this study, a cost-benefit analysis is warranted to compare the cost of operating on patients later *versus* earlier before classical symptoms manifest.

However, only a minor part of the variation in incidence could be explained by inequality associated with age, sex or socioeconomic factors. When comparing high- and low-incidence regions, a specific effect of education was observed, driven by elderly patients in the high-incidence regions. This association with education was not seen in the low-incidence regions or for age under 70. Patients of African ethnicity were overrepresented and were characterized by the removal of larger parathyroid adenomas in high-incidence regions alone. The confounding effect of patients migrating with untreated PHPT from countries with limited healthcare supply should be considered, although a genetic predisposition might also be a contributing factor. No effect of individual income on surgical management was observed.

As expected, patients treated with PTX had a higher risk of developing kidney failure, kidney stones and osteoporosis than controls. This risk increased manifold with time from 5 years to 1 year before surgery. The prevalence of registered kidney stones was significantly higher in regions with a low incidence of PTX (9.5% *versus* 5.3% the year before surgery, *P* < 0.001). Musculoskeletal diagnoses, osteoporosis and NSAID distribution were also significantly more frequent in low-incidence regions 5 years and 1 year before surgery. These findings probably reflect differences in diagnostic intensity and approach to surgical treatment of PHPT, but this study does not allow the definition of any optimal incidence of PTX. It is possible that the correct evidence-based incidence of PTX should be somewhere in between the extremes. Seib *et al.* reported that only 29% of a cohort of patients with PHPT covered by the Medicare beneficiaries, diagnosed between 2006 and 2016 and fulfilling the consensus guideline criteria for PTX, received surgical treatment^27^. In this case-control study of patients with PHPT already selected for PTX, 79 and 68% of patients in regions with low and high incidence of PTX fulfilled well-established criteria for surgery respectively.

Cardiovascular diagnoses were overrepresented among patients with PHPT selected for PTX. Since hypertension is mainly treated by primary care physicians and thus suboptimally registered in the NPR, surveillance bias may lead to falsely low numbers among the controls. However, prescriptions of antihypertensives, diuretics and beta-blockers were all significantly higher in the PTX group. The prevalence of filled prescriptions of antihypertensives increased by 18.1% between 5 years and 1 year before the index compared with 10.4% for control, resulting in an OR of 2.06 (95% c.i. 1.96 to 2.17). Arrhythmias and stroke were more common in the PTX group (OR 2.39, 95% c.i. 2.26 to 2.65 and OR 1.59, 95% c.i. 1.23 to 2.06 respectively). Also, there was a three-fold increase in the preoperative prevalence of pancreatitis for patients with PHPT selected for PTX from years 5 to 1. As recently reported, an overall increased risk of psychiatric illness before surgery and increased use of antidepressants, sedatives and anxiolytics was noted^28^.

Patients in low-incidence regions had larger parathyroid adenomas removed, indicating a longer disease duration. Thus, patients in the low-incidence regions had a higher clinical threshold for surgery, as represented by a more considerable preoperative prevalence of both classical and non-classical symptoms, drug prescriptions, calcium levels and adenoma weights. Finally, there was a significant increase in the prevalence of cardiovascular diagnoses: hypertension, arrhythmias and stroke in the entire group of patients with PHPT selected for PTX despite relatively low calcium levels.

Overall, there was a significant difference in healthcare supply and demand between low- and high-incidence regions. This difference was seen for median waiting times for PTX and the number of visits to outpatient clinics. Still, it did not explain the large discrepancy in the utilization of PTX between the regions. To assess geographical differences in care-seeking behaviour, the prevalence of diagnoses and drug prescriptions was compared between controls in high- *versus* low-incidence regions. Controls in high-incidence regions had a slightly higher prevalence of co-morbidities, as shown in *Table 3*. This can be interpreted as a sign of a larger healthcare supply in these regions if it is assumed that patient health is not associated with the residency location. This difference in baseline health for the two types of regions was small (under 1% absolute prevalence difference for most diagnoses).

The strength of this population-based study is the reliable registry data where patient co-morbidities are extensively covered. The main weakness is the inclusion of patients already selected for PTX, limiting the generalizability of undiagnosed or conservatively treated patients with PHPT to the entire population. It is likely that the majority of diagnosed but non-operated on patients with PHPT are monitored in primary care and thus not included in the NPR, nor in any national register. Increased consumption of antidepressive medication and benzodiazepines among patients with PHPT selected for surgery was reported earlier^28^. Surveillance bias must be considered when comparing the prevalence of diagnoses between patients and controls. The Swedish Prescribed Drug Register is an important asset, covering all prescribed drugs obtained from pharmacies. By including patients from validated and overlapping registries, we can assume that almost all patients undergoing surgery for PHPT in the interval were included, and no substantial selection bias was created. Having the NPDR with information on drug prescriptions decreased the information bias created by the lack of ICD registration in primary care. Since PTX patients have had their diagnostic work-up through specialized care, it can be assumed that the prevalence of co-morbidities is accurately measured in this group. The observed differences in the prevalence of co-morbidities and prescribed medications between PTX patients and controls should be interpreted cautiously because of information bias (that is co-morbidities lead to PHPT diagnosis, which leads to co-morbidity registered in NPR). This bias is most profound for diagnoses mainly treated in primary care and is offset to some extent by including information on prescribed medications.

Adenoma weights were missing in 38% of patients from low-incidence regions compared with 32% in the high-incidence regions. This could introduce a bias in either direction when comparing the two types of regions. However, after applying multiple imputation the differences between regions were virtually unchanged, and the difference in adenoma weight and calcium level means between regions with low and high incidence of PTX remained statistically significant.

Finally, a limitation of the study was the absence of registered primary care diagnoses. Data from primary care are needed to create a complete picture of how patients in Sweden are diagnosed and treated for PHPT and other conditions.

## Supplementary Material

zrad154_Supplementary_Data

## Data Availability

Data is not available to other researchers due to ethical and legal restrictions. R code for data tidying, statistical methods, graphs and pictures are available from the corresponding author upon request.
