# Supplementary material for: Regional variations in the management of primary hyperparathyroidism in Sweden: population-based case-control study
Source: BJS Open. 2024 Feb 7;8(1):zrad154. doi: 10.1093/bjsopen/zrad154 (PMC10848304; doi:10.1093/bjsopen/zrad154)
Supplement: zrad154_Supplementary_Data [file zrad154_supplementary_data.docx]

**Title**

Regional variations in the management of primary hyperparathyroidism in Sweden:

population-based case-control study

# Authors

David Thorsteinsson^1,2^, Fredrik Granath^3^, Robert Bränström^1,2^, Anna Koman^1,2^, Jan Zedenius^1,2^, Inga-Lena Nilsson^1,2.^

- - - 1. Department of Breast, Endocrine Tumours and Sarcoma, Karolinska University Hospital, Stockholm, Sweden.
      2. Department of Molecular Medicine and Surgery, Karolinska Institutet, Stockholm, Sweden.
      3. Department of Medicine Solna, Division of Clinical Epidemiology, Karolinska Institutet, Stockholm, Sweden

**Corresponding author**

David Thorsteinsson

Endocrine surgical Unit

Department of Molecular Medicine and Surgery

Karolinska Institutet

Karolinska University Hospital,

Eugeniavägen 3, 171 76 Stockholm, Sweden

E-mail: david.thor.thorsteinsson@ki.se

**ORCID ID** 0009-0004-1043-1733

**Supplementary Materials – Index**

| **Supplementary Tables**  Supplementary Table Legends | *Page 2* |
| --- | --- |
| Supplementary Table 1 | *Page 3* |
| Supplementary Table 2 | *Page 4* |
| Supplementary Table 3 | *Page 5* |
|  |  |
|  |  |
|  |  |
|  |  |
|  |  |

**Supplementary Tables**

# Supplementary Table legends

*Supplementary Table 1. ICD-10 and ATC Index codes for diagnoses and prescribed drugs*

*Supplementary Table 2. Diagnoses and filled prescriptions for patients and controls one and five years before the index date.*

*Supplementary Table 3. Socioeconomic factors of patients by region*

| **Supplementary Table 1. ICD-10 and ATC Index codes** | |
| --- | --- |
| **Variable** | **ICD-10 and/or ATC Index Codes** |
| Antidiabetics | A10 |
| Anxiolytics | N05B |
| Arrythmia | I44-52 |
| Diuretics | C03 |
| Fragility fractures | S12,22,32,42,52,72 and fall on same level (W00,01,03) |
| Gallstones | K80 |
| Hypertension +/- antihypertensives | I10 +/- C02,08,09 |
| Kidney failure | N17-19 |
| Kidney stones | N20-22 |
| Musculoskeletal diagnoses | M00-99 |
| Myocardial infarction | I21 |
| NSAID medication | M01 |
| Osteoporosis +/-bisphosphonates | M80-82 +/- M05B |
| Pancreatitis | K85 |
| Proton pump inhibitors | A02BC |
| Psychiatric illness | F06-99 |
| Sedatives | N05C |
| Stroke | I60 |

| **Supplementary Table 2. Diagnoses and filled prescriptions one and five years before parathyroidectomy (PTX)** | | | | | | | | | | | | | |  |
| --- | --- | --- | --- | --- | --- | --- | --- | --- | --- | --- | --- | --- | --- | --- |
| **Variable (ICD, ATC)** | **One year before PTX** | | | | |  | | **Year five before PTX** | | | | | |  |
|  | **OR** | **95% CI** | **Patients (%)** | **Controls (%)** |  | | **OR** | | **95% CI** | **Patients (%)** | | **Controls (%)** | |  |
| Kidney stones | 19.6 | (16.9-22.8) | 5.9 | 0.3 |  | | 4.2 | | (3.3-5.3) | 1.1 | | 0.3 | |  |
| Kidney failure | 4.9 | (4.2-5.7) | 2.9 | 0.6 |  | | 3.4 | | (2.6-4.4) | 0.8 | | 0.2 | |  |
| Osteoporosis * | 4.6 | (4.3-5.0) | 12.0 | 3.2 |  | | 1.5 | | (1.3-1.8) | 2.9 | | 2.0 | |  |
| Fractures § | 1.9 | (1.6-2.2) | 2.0 | 1.1 |  | | 1.2 | | (0.9-1.5) | 0.96 | | 0.82 | |  |
| Hypertension † | 2.4 | (2.3-2.6) | 45.4 | 28.5 |  | | 1.6 | | (1.5-1.7) | 24.3 | | 18.0 | |  |
| Arrythmia | 2.4 | (2.2-2.7) | 5.9 | 2.7 |  | | 1.4 | | (1.2-1.6) | 2.1 | | 1.5 | |  |
| Diuretics (C03) | 1.6 | (1.5-1.7) | 19.4 | 13.9 |  | | 1.5 | | (1.4-1.6) | 13.1 | | 9.4 | |  |
| Stroke | 1.6 | (1.2-2.1) | 0.8 | 0.5 |  | | 0.7 | | (0.5-1.1) | 0.2 | | 0.3 | |  |
| Myocardial infarction | 1.2 | (0.8-1.6) | 0.4 | 0.4 |  | | 0.8 | | (0.5-1.3) | 0.2 | | 0.3 | |  |
| Antidiabetics | 1.0 | (1.0-1.1) | 7.6 | 7.3 |  | | 1.0 | | (0.9-1.1) | 4.6 | | 4.6 | |  |
| Psychiatric illness | 2.3 | (2.7-2.5) | 9.0 | 4.1 |  | | 1.3 | | (1.2-1.5) | 4.0 | | 3.1 | |  |
| Sedatives | 1.4 | (1.3-1.5) | 12.6 | 9.2 |  | | 1.1 | | (1.0-1.2) | 7.1 | | 6.3 | |  |
| Anxiolytics | 1.4 | (1.3-1.5) | 20.0 | 15.3 |  | | 1.2 | | (1.1-1.3) | 12.3 | | 10.7 | |  |
| Pancreatitis | 5.5 | (3.6-8.3) | 0.4 | 0.1 |  | | 2.1 | | (1.1-4.1) | 0.1 | | 0.1 | |  |
| Gallstones | 2.4 | (1.9-3.0) | 1.0 | 0.4 |  | | 2.0 | | (1.6-2.7) | 0.7 | | 0.4 | |  |
| Proton pump inhibitors | 1.8 | (1.7-1.9) | 25.0 | 16.1 |  | | 1.4 | | (1.3-1.5) | 14.2 | | 10.7 | |  |
| *ICD-diagnosis and/or treatment with drugs affecting bone mineralisation, ATC/DDD M05B | | | | | | | | | | | | | |  |
| § Fracture ICD codes S12, S22, S32, S42, S52, S72 and fall on same level (W00, W01, W03). | | | | | | | | | | |  | |  |  |
| † ICD-diagnosis and/or treatment with antihypertensives (calcium channel blockers or agents acting on the renin angiotensin system, ATC/DDD Index C02, C08 and C09) | | | | | | | | | | | | | |  |
|  |  |  |  |  |  |  |  |  |  |  |  |  |  |  |

| **Supplementary Table 3. Socioeconomic factors of patients by region** | | | | | | | |  |
| --- | --- | --- | --- | --- | --- | --- | --- | --- |
| **Variable** | **Low incidence regions** | |  | **High incidence regions** | | |  | |
|  | **n= 1 161** | |  | **n = 7 465** | | |  | |
|  | **OR** | **95%CI** |  | **OR** | **95% CI** |  | | |
| **Education** |  |  |  |  |  |  | | |
| Primary | 1 | - |  | 1 | - |  | | |
| Secondary | 0.97 | 0.83-1.14 |  | 1.08 | 1.02-1.15 |  | | |
| Tertiary | 1.04 | 0.87-1.24 |  | 1.10 | 1.03-1.18 |  | | |
| **Ethnicity** |  |  |  |  |  |  | | |
| Nordic Countries | 1 |  |  | 1 |  |  | | |
| Africa | 0.94 | 0.37-2.36 |  | 1.49 | 1.17-1.88 |  | | |
| Asia | 0.78 | 0.47-1.28 |  | 0.95 | 0.83-1.09 |  | | |
| Europe | 1.05 | 0.72-1.52 |  | 0.96 | 0.87-1.06 |  | | |
| Americas | 1.75 | 0.51-6.04 |  | 1.09 | 0.70-1.72 |  | | |
| Oceania | 0.01 | NA |  | 0.79 | 0.10-6.11 |  | | |
| Unknown | NA | NA |  | 0.05 | NA |  | | |
| **Total taxed income kSEK** | **Cases** | **Controls** | **P*** | **Cases** | **Controls** | **P*** | | |
| Individual - mean (SD) | 1 993(1 188) | 2 036(1 809) | 0.3 | 2 324(3 480) | 2 282(3 717) | 0.3 | | |
| Family - mean (SD) | 3 633(2 598) | 3 689(3 400) | 0.5 | 4 148(4 712) | 4 107(5 464) | 0.5 | | |
| * Welch´s two sample t-test |  |  |  |  |  |  | | |
